# Optically-Induced Symmetry Switching in a Reconfigurable Kagome Photonic Lattice: From Flatband to Type-III Dirac Cones

**DOI:** 10.3390/nano12183222

**Published:** 2022-09-16

**Authors:** Qingsong Yu, Zhenzhi Liu, Dawei Guo, Shun Liang, Yanpeng Zhang, Zhaoyang Zhang

**Affiliations:** Key Laboratory for Physical Electronics and Devices of the Ministry of Education & Shaanxi Key Lab of information Photonic Technique, School of Electronic Science and Engineering, Faculty of Electronic and Information Engineering, Xi’an Jiaotong University, Xi’an 710049, China

**Keywords:** flatband, Dirac cones, Kagome photonic lattice, electromagnetically induced transparency

## Abstract

We demonstrate the transition of band structure from flatband to type-III Dirac cones in an electromagnetically induced Kagome photonic lattice generated in a three-level Λ-type ^85^Rb atomic configuration both experimentally and theoretically. Such instantaneously reconfigurable Kagome photonic lattice with flatband is “written” by a strong coupling field possessing a Kagome intensity distribution, which can modulate the refractive index of atomic vapors in a spatially periodical manner under electromagnetically induced transparency. By introducing an additional one-dimensional periodic coupling field to cover any one set of the three inequivalent sublattices of the induced Kagome photonic lattice, the dispersion-less energy band can evolve into type-III Dirac cones with linear dispersion by easily manipulating the intensity of the one-dimensional field. Our results may pave a new route to engineer in situ reconfigurable photonic structures with type-III Dirac cones, which can act as promising platforms to explore the underlying physics and beam dynamics.

## 1. Introduction

In the last decade, research on photonic lattices with various band structures have attracted considerable interest [1,2,3], among which the photonic structures with flatband [4,5] or Dirac cones [6,7] promptly acquired special notice due to their unique properties. The flatband is a completely dispersionless energy band in the whole Brillouin zone, and its eigenmodes are highly degenerate [8]. Lights that excite flatband modes are strongly localized and have diffraction-free transmission properties [9,10]. Therefore, those photonic lattices with flatbands, such as Lieb and Kagome lattices, are widely used for research on beam localization [11], lossless optical information [12], and distortion-free image transmission [13,14], to name a few. In contrast to the flatband, Dirac cones exhibit linear dispersion, and have been extensively studied in honeycomb lattices (i.e., photonic graphene) [15,16,17,18]. Beams travelling through photonic structures with Dirac cones can lead to interesting phenomena such as cone diffraction [19,20,21], Klein tunneling [22,23], and Zitterbewegung [24], etc. Moreover, according to the different geometries of the Fermi surface, there exist three types of Dirac cones [25]. The type-I Dirac cones in honeycomb lattices are characterized by their linear dispersion in all directions in *k* space and point-like on the Fermi surface [26]. Different from the type-I ones, the type-II Dirac cones appear as a pair of crossing lines on the Fermi surface and exhibit non-isotropic transport properties, which are extremely attractive for studies of nontrivial topological phenomena [27,28,29]. The type-III Dirac cones, emerging from the transition from type-I to type-II [25,30], combine flatband and linear dispersions and appear as a line on the Fermi surface. This kind of Dirac cone has been predicted to realize a black-hole event horizon in condensed matter [31,32].

The commonly used methods to generate photonic lattices with specific band structures include laser direct writing [33] and optical induction in photorefractive crystals [34], etc. All the three types of Dirac cones have been realized by the aforementioned methods [25,35,36]. Recently, by taking advantages of the instantaneous tunability of the atomic medium, the optical induction method is applied in coherently-prepared atomic vapors to construct various one- and two-dimensional electromagnetically induced photonic lattices [37,38,39,40,41,42], which can exhibit desired band structures by easily adjusting the laser parameters to change the refractive index profiles. Interesting beam dynamics such as particle-like behaviors of optical vortex [43] and edge-state solitons [44] have been investigated by easily changing the band structures in such in situ tunable platforms. So far, reconfigurable Dirac photonic structures based on atomic coherence are mostly limited in the type-I Dirac cones in photonic graphene [18,45]. The experimental demonstration of the other two types of Dirac cones has not been reported in electromagnetically induced photonic lattices probably due to the difficulty of generating a required photonic structure in atomic vapors.

In this paper, we propose a simple experimental approach for constructing type-III Dirac cones by modulating the band structure in a Kagome photonic lattice based on atomic coherence. In the experiment, we first construct the Kagome photonic lattice with a flatband in three-level Λ-type ^85^Rb vapors under electromagnetically induced transparency (EIT) [46], and then a one-dimensional periodic coupling field is introduced to cover one set of the three inequivalent sublattices of the formed Kagome photonic structure. As a result, the original flatband is switched to type-III Dirac cones, which is verified by the change in the output diffraction patterns of the incident probe beam passing through the induced lattices. Furthermore, we explore the evolution of formed type-III Dirac cones under different experimental parameters, and a comprehensive theoretical simulation is also given to support the observations. The current work provides a new method to generate in situ reconfigurable photonic structures with type-III Dirac cones and a new platform to investigate the underlying beam dynamics around the type-III Dirac cones.

## 2. Experimental Scheme

**Figure 1:**
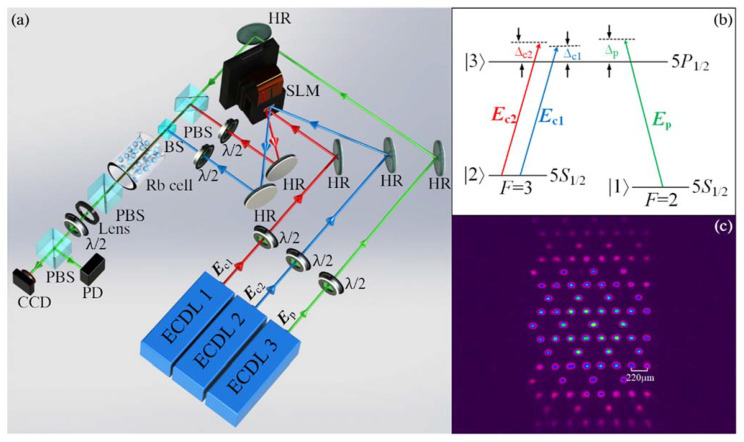
(**a**) Experimental setup. The coupling beam ***E***_c1_ from ECDL1 is modulated into a Kagome intensity distribution by a SLM. The other coupling beam, ***E***_c2_ from ECDL2, is transformed into a one-dimensional periodic field by the same SLM. The probe field ***E***_p_ from ECDL3 co-propagates with two coupling fields, and its output pattern is imaged onto the CCD camera through an imaging lens. The spot size of the used beams is ~ 3.5 mm. The powers of ***E***_c1_, ***E***_c2_, and ***E***_p_ are 20 mW, 10 mW, and 1 mW, respectively. The divergence angles of the beams from the adopted ECDLs in the *x* and *y* directions are ~0.89 mrad and ~1.81 mrad, respectively. ECDL: external cavity diode laser; λ/2: half-wave plate; HR: high-reflectivity mirror; SLM: spatial light modulator; PBS: polarization beam splitter; BS: beam splitter; and PD: photodiode detector. CCD: charge-coupled device. (**b**) The three-level Λ-type ^85^Rb atomic structure. (**c**) The observed transmitted probe pattern with only the Kagome coupling field turned on.

The experimental setup is shown in Figure 1a. The coupling beams ***E***_c1_ and ***E***_c2_ emitted from two external cavity diode lasers (ECDL1 and ECDL2) are respectively launched to the left and right half of the screen of a phase-controlled spatial light modulator (SLM). The screen of the SLM is divided into two independent regions, and the two input Gaussian beams can be modulated simultaneously. By loading appropriate phase diagrams on the left and right regions of the screen, the amplitude and phase of the two input beams can be modulated to establish the required coupling fields with respective Kagome and one-dimensional periodic intensity profiles. The output two periodic fields are reflected into the atomic medium by the screen of the SLM together with other optical devices. The vertically polarized Kagome coupling field ***E***_c1_ (frequency ω_c1_, periodicity 220 μm) and field ***E***_c2_ (frequency ω_c2_, periodicity 380 μm) travel through the ^85^Rb vapor cell along the *z* direction. The vapor cell is 2.5 cm in diameter and 3 cm in length and is wrapped up in the *µ*-metal sheet to isolate it from the influence of external magnetic fields. The medium is heated to 363 K by winding a heating tape on the cell to provide an atomic density of N ≈ 6 × 10^12^ cm^−3^. The weak Gaussian probe field ***E***_p_ (frequency ω_p_, horizontal polarization) from ECDL3 is injected into the vapor cell in the same direction as the coupling fields. As a result, a three-level atomic configuration in Figure 1b is prepared to generate EIT. The probe field ***E***_p_ connects the transition between the excited level 5P_1/2_ (|3⟩) and one ground level, 5S_1/2_, F = 2 (|1⟩), while the two coupling beams drive the same transition from the other ground level, 5S_1/2_, F = 3 (|1⟩) to 5P_1/2_ but with different frequency detuning. The output wavelength of the three ECDLs is around 795.0 nm. Here, the detuning Δ_p_ (Δ_c1_, Δ_c__2_) is defined as the difference between the frequency of ***E***_p_ (***E***_c1_, ***E***_c__2_) and the gap between the two energy levels it drives. By properly setting the laser frequency, the two-photon resonance conditions Δ_p_ − Δ_c1_ = 0 and Δ_p_ − Δ_c2_ = 0 can be achieved to generate two partially overlapped EIT windows. Due to the different polarizations of the probe and coupling fields, a polarization beam splitter (PBS) is placed next to the rear surface of the vapor cell to filter out the two coupling fields, and only the probe beam is detected. The output probe field is divided into two parts by a PBS together with a half-wave plate. One part is imaged onto a charge-coupled device (CCD) camera by an imaging lens to observe the spatial image at the output plane of the cell, while the remaining part is received by the photodiode detector to monitor the transmitted spectrum with EIT.

With only the probe field and Kagome coupling field turned on, the output probe beam can exhibit discrete patterns in Kagome geometry around the EIT window generated by ***E***_c1_, which is responsible for “writing” a Kagome photonic lattice by modulating the refractive index, in a spatially periodic manner inside the vapor cell. The observed output probe image is shown in Figure 1c. Further, with the one-dimensional coupling field ***E***_c2_ introduced, two EIT windows will occur simultaneously when both two-photon resonance conditions Δ_p_ − Δ_c1_ = 0 and Δ_p_ − Δ_c2_ = 0 are satisfied. This means that both of the two coupling fields can exert modification on the spatial refractive index and the behavior of the probe field will be governed by a combined effect of the two periodic fields. By carefully elaborating the spatial alignment of the two coupling fields to make the one-dimensional field cover one set of sublattices on the Kagome photonic lattice accurately through the whole medium along *z*, the original flatband can evolve to type-III Dirac cones.

## 3. Results and Discussions

### 3.1. Theoretical Analysis of Band Structure

Theoretically, the total refractive index n can be described as n = $\sqrt{1+\chi }$ ≈ 1 + χ/2 in an EIT atomic medium, where the susceptibility χ can control the dynamical behaviors of the incident probe field. In consideration of n = n_0_ + Δn (n_0_ ≈ 1 represents the background index of the atomic gas), the variation of the refractive index caused by the EIT effect can be expressed as Δn ≈ χ/2. According to the density-matrix equations for the three-level atomic configuration [47], one can obtain χ = (2Nμ_31_/ε_0_E_p_) × ρ_31_, where N is the atomic density, μ_ij_ (i, j = 1, 2, 3) and ρ_ij_ are the dipole moment and the density-matrix element corresponding to the transition of |i⟩ → |j⟩, respectively, E_p_ is the electric-field intensity of the probe field ***E***_p_, and ε_0_ is the permittivity of vacuum. Consequently, the susceptibility in spatial modulation can be expressed as [23]:

(1) $$\chi =\frac{\mathrm{iN}{\left|\mu_{31}\right|}^{2}}{\hbar \varepsilon_{0}}\times {\left(\left(\Gamma_{31}+i\Delta_{p}\right)+\frac{{\left|\Omega_{c1}\right|}^{2}}{\Gamma_{32}+i\left(\Delta_{p}-\Delta_{c1}\right)}+\frac{{\left|\Omega_{c2}\right|}^{2}}{\Gamma_{32}+i\left(\Delta_{p}-\Delta_{c2}\right)}\right)}^{-1},$$

where Ω_c1_ (Ω_c2_) is the Rabi frequency of the coupling field ***E***_c1_ (***E***_c2_) and |Ω_c1_|^2^ and |Ω_c__2_|^2^ represent the intensity profile of corresponding beams, Γ_ij_ is the decay rate between two states of |i⟩ and |j⟩, and ħ is the reduced Planck constant. Here, the Rabi frequency is defined as Ω_c1(c2)_ = μ_32_E_c1(c2)_/ħ. According to Equation (1), the refractive index as well as band structures can be effectively controlled by easily changing the parameters such as frequency detuning and beam intensity.

**Figure 2:**
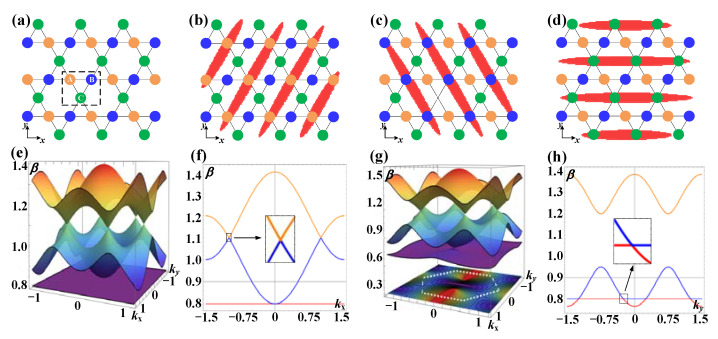
(**a**) The geometric sketch of a Kagome lattice. Three inequivalent sublattices (A, B, and C) are indicated by orange, blue, and green in the dotted box. (**b**–**d**) depict the spatial arrangements of the periodic one-dimensional coupling field (red fringes) that covers different sets of sublattices in the Kagome lattice inside the ^85^Rb cell in *x-y* plane. (**e**) Band structure of the Kagome lattice corresponding to (**a**). (**f**) The flatband and traditional type-I Dirac cones appear at *β* = 0.8 and 1.1, respectively, in the cross-sectional graph of (**e**). (**g**) The band structure of the modulated Kagome lattice corresponding to (**d**). (**h**) Cross-sectional graph of (**g**), and the type-III Dirac points appear.

When the one-dimensional coupling field ***E***_c2_ is blocked, namely, term |Ω_c2_|^2^ is 0 in Equation (1), the susceptibility can exhibit a Kagome profile. There are three sets of inequivalent sublattice (A, B, and C) in the Kagome photonic lattice [48], as shown in Figure 2a. With any one set of the sublattice covered by the added one-dimensional coupling field (|Ω_c2_|^2^ ≠ 0), as shown in Figure 2b–d, the resulted susceptibility as well as the corresponding band structures can be different from the original Kagome structure. According to the tight-binding approximation method [49], the calculated two-dimensional band structures of the Kagome photonic lattice with |Ω_c2_|^2^ = 0 and |Ω_c2_|^2^ ≠ 0 are shown in Figure 2e,g, respectively. In order to analyze them more intuitively, the corresponding cross-sectional graphs are given in Figure 2f,h. Obviously, the original Kagome photonic lattice has a flatband (*β* = 0.8). With the introduction of the modulation of ***E***_c2_, the flatband disappears and new Dirac cones [marked by the dashed box in Figure 2h] are formed. Compared with the traditional Dirac cones characterized by a linear dispersion in all directions in *k* space [the inset in Figure 2f], the new formed Dirac cones are strongly tilted [the inset in Figure 2h], which are exactly the type-III Dirac cones that have a flatband and a linear dispersion, and the Fermi surface is a line [25]. Due to the rotation symmetry of the Kagome lattice, regardless of which set of sublattice is covered, the type-III Dirac cones are formed, and the only difference is the direction [50,51].

### 3.2. Theoretical Simulation of Beam Dynamics

**Figure 3:**
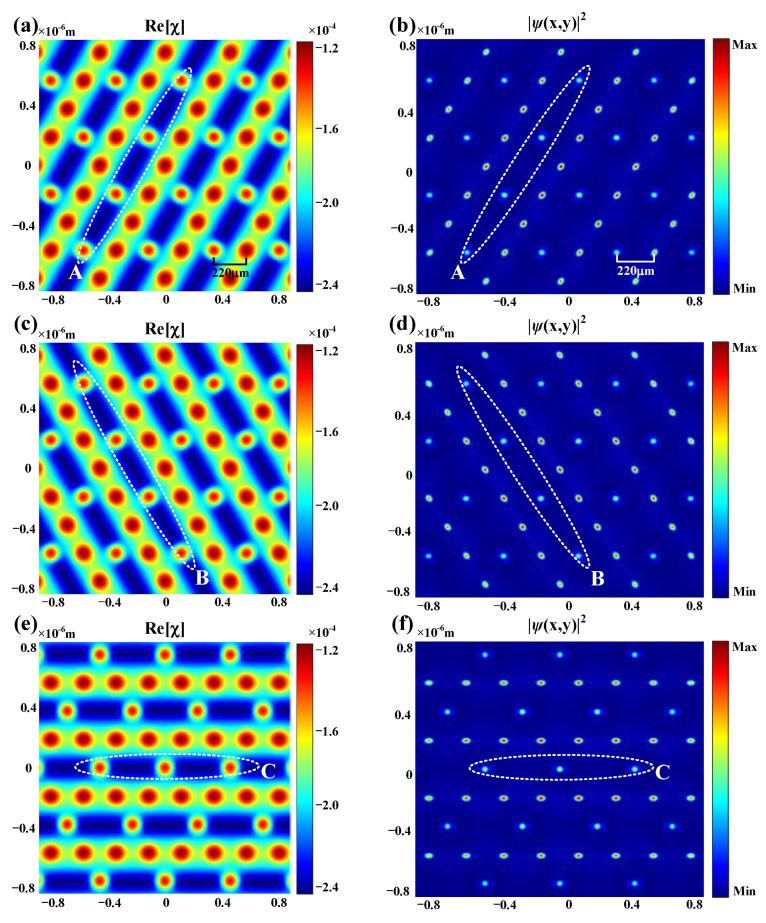
Theoretical simulation of the real part of the susceptibility Re[χ] when the periodic one-dimensional coupling field covers the (**a**) A sublattices, (**c**) B sublattices, and (**e**) C sublattices of the Kagome photonic lattice. The corresponding intensity distributions of the output probe field are given in (**b**,**d**,**f**), respectively (the dotted ellipse in each panel represents the covered sublattices). The following parameters are used in the theoretical simulation: Δ_p_ = 100 MHz, Δ_c1_ = 110 MHz, Δ_c2_ = 90 MHz, the atomic density is N = 6 × 10^12^ cm^−3^, Ω_c1_ = 125 MHz, and Ω_c2_ = 80 MHz.

Under the condition of Δ_p_ = 100 MHz, Δ_c1_ = 110 MHz, and Δ_c2_ = 90 MHz, according to Equation (1), the distributions of the real part Re[χ] are obtained as Figure 3a,c,e when the periodic one-dimensional coupling field covers different set of sublattices. It is clear that the susceptibility of the covered sites is different from that of the uncovered sites, and such a difference can lead to distinct dynamic behaviors in corresponding channels. The propagation of the probe field in such discrete systems are guided by the following Schrodinger-like paraxial equation [45]:

(2) $$i\frac{\partial \psi \left(x,y,z\right)}{\partial z}=-\frac{1}{2k_{0}}\left(\frac{\partial^{2}}{\partial x^{2}}+\frac{\partial^{2}}{\partial y^{2}}\right)\psi -\frac{k_{0}}{n_{0}}\Delta n\left(x,y\right)\psi ,$$

where ψ is the probe-field envelope ***E***_p_, k_0_ is the wavenumber, z is the propagation distance, and Δn(x,y) is the optically induced potential well via atomic coherence dominated by the combined modulation of two EIT windows. According to Equation (2), the intensity profiles of the output probe field in the cases of covering different sublattices of the Kagome lattice can be obtained and are shown in Figure 3b,d,f, which correspond to Figure 3a,c,e respectively. On one hand, considering the Kagome refractive index pattern, the output probe beam shows the same discretized structure as the susceptibility, in which the sites with larger values can “attract” the energy of the beam energy during propagation and act as waveguide channels. On the other hand, the output intensity of the probe beam at the covered sections is suppressed, which is obviously due to the decrease in susceptibility after ***E***_c2_ modulation in the corresponding regions.

### 3.3. Experimental Results and Discussions

**Figure 4:**
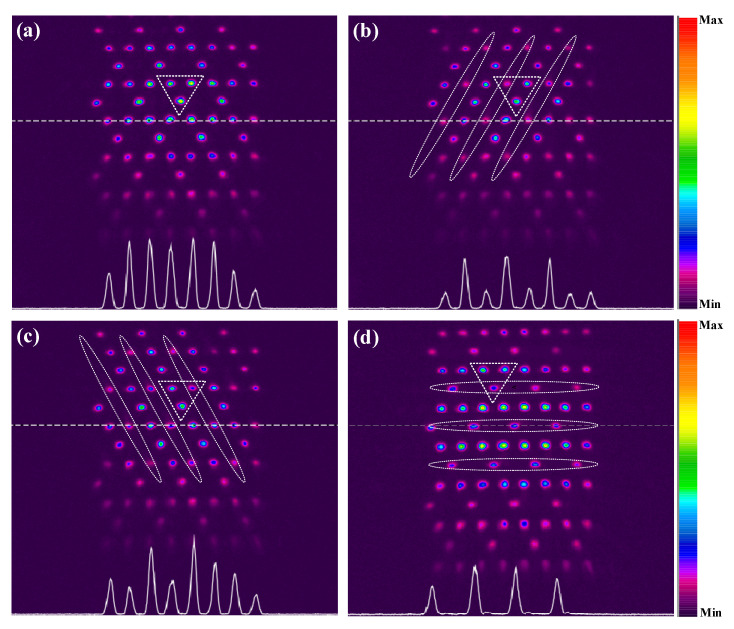
(**a**) Observed output probe patterns with only the Kagome coupling field on. (**b**–**d**) The one-dimensional coupling field covers A, B, and C sublattices in the induced Kagome lattice. The dotted ellipses denote the position of the introducing coupling field ***E***_c1_ and the dashed triangle in each picture represents the unit cell of the Kagome photonic lattice. The two-photon detuning of two coupling fields ***E***_c1_ and ***E***_c2_ is fixed at δ_c1_ = Δ_p_ − Δ_c1_ = −10 MHz and δ_c2_ = Δ_p_ − Δ_c1_ = 10 MHz, respectively, with Δ_p_ = 100 MHz. The power of the probe field is P_p_ = 1 mW, and the power of the coupling fields ***E***_c1_ and ***E***_c2_ are P_c1_ = 20 mW and P_c2_ = 10 mW, respectively.

Figure 4a shows the measured output probe pattern without the one-dimensional coupling field ***E***_c2_ around the EIT window generated by the Kagome coupling field ***E***_c1_. With the two-photon detuning set as δ_c1_ = Δ_p_ − Δ_c1_ = −10 MHz at probe detuning Δ_p_ = 100 MHz, a clear discrete pattern in Kagome geometry is observed at the output plane of the vapor cell. Then we introduce the ***E***_c2_ field to generate the other EIT window, which is partially overlapped with the first EIT to ensure that the probe field experiences joint modulation of the two coupling fields. By adjusting the parameters of the Damann grating to control the period and direction of the coupling field ***E***_c2_, we can realize three different spatial arrangements [Figure 2b–d] to modulate each set of sublattices of the Kagome photonic structure induced inside the ^85^Rb cell. With the two-photon detuning δ_c2_ = Δ_p_ − Δ_c2_ being 10 MHz, the evolution of the output probe patterns with different sublattices covered are shown in Figure 4b–d, which agree well with the simulation in Figure 3.

The change in the unit cell (the dashed triangle in each panel in Figure 4) of the Kagome photonic lattice indicates the modulation of the band structure. For the case of without modulation, the three sublattices in the unit cell have the same intensity, as shown in Figure 4a. After introducing the coupling field ***E***_c1_ (whose location is marked by dotted ellipses) to cover each set of sublattices, the refractive index as well as the band structure will be modulated accordingly. As a result, it can be seen that the intensity of the covered sublattice in the unit cell is weakened. The white curve at the bottom of each picture in Figure 4 shows the intensity of the region marked by a dashed line. This white dashed line and the intensity curve come from the software of the CCD camera itself.

**Figure 5:**
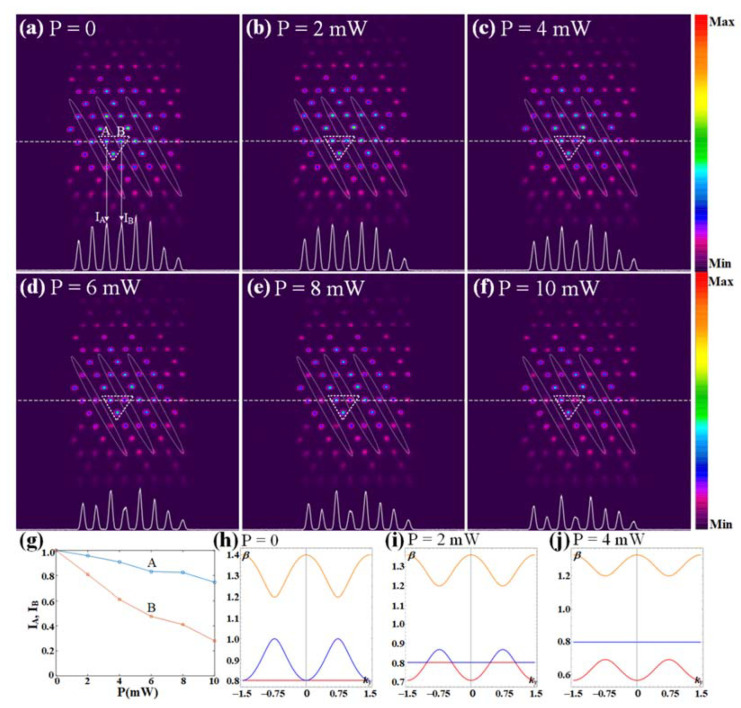
(**a**–**f**) Evolution of the output probe field with the power of the coupling field ***E***_c2_ increases from 0 to 10 mW. The B sublattices of the Kagome lattices are covered by ***E***_c2_, whose position is indicated by dotted ellipses. The two-photon detuning of two coupling fields ***E***_c1_ and ***E***_c2_ is fixed at δ_c1_ = −10 MHz and δ_c2_ = 10 MHz respectively, with Δ_p_ = 100 MHz. The power of the probe field ***E***_p_ and the Kagome coupling field ***E***_c1_ are 1mW and 20 mW, respectively. (**g**) The dependences of intensity at A and B sites [marked by two arrows in (**a**)] on the power. (**h**–**j**) The evolution of the type-III Dirac band structures with increasing the power of the one-dimensional coupling field ***E***_c2_, and the power corresponding to (**h**–**j**) are 0, 2 mW, and 4 mW, respectively.

According to Equation (1), the structured Rabi frequency is a critical parameter that determines the spatial refractive index distribution and is directly related to the power of laser beams. A benefit from the easily accessible tunability of the electromagnetically induced optical lattices is that we can explore the dynamics of light that reflects the variation of the band structure by adjusting the laser power [52]. As such, we demonstrate the modulation effect of the introduced coupling field ***E***_c2_ on the band structure by adjusting its power. Firstly, we fix the power of the Kagome-lattice coupling field ***E***_c1_ at 20 mW, which allows us to observe a Kagome pattern [Figure 5a] with high contrast at the output plane of the cell. Then, we increase the power of ***E***_c2_ to 10 mW gradually, and the evolution of the output probe is shown in Figure 5b–f. The coupling field ***E***_c2_ (denoted by dotted ellipses) covers the B sublattices of the Kagome lattice. We focus on the unit cell marked by the dashed triangle Figure 5a–f and find that the intensity of B sites on output patterns is getting smaller with the growing of the power, while the other two sublattices become slightly weaker. The intensity contrast between the modulated B sites and the other two sites is the most obvious at 10 mW, as depicted in Figure 5f. In addition, based on the intensity curves generated by the software of the CCD camera in Figure 5a–f, we plot the dependence of the intensity at A and B sites on the power of the one-dimensional coupling field, as shown in Figure 5g. This proves the modulation of the introducing coupling field on the Kagome photonic lattice is effective and modulation depth can increase with power. The intensity weakening of the sublattice B is due to the refractive index n declining at the corresponding position. Since the refractive index is proportional to the real part of the susceptibility, when the Rabi frequency Ω_c2_ increases with ***E***_c2_ power, the real part of the susceptibility Re[χ] will decrease, which can be deduced from Equation (1). The slight intensity changes of the A and C sites can be understood as the energy exchange caused by the coupling between neighboring waveguide channels. In detail, as the ***E***_c2_ power increases, the energy in B channels decreases dramatically, and the energy in A and C channels is coupled into B during the propagation. Finally, we use the tight-binding method to qualitatively simulate the band structures at different powers of the one-dimensional coupling field ***E***_c2_, as depicted in Figure 5h–j. With the power (Rabi frequency) increasing, the type-III Dirac cones will gradually disappear. 

## 4. Conclusions and Discussions

In summary, the immediate symmetry switching from flatband to type-III Dirac cones in a reconfigurable Kagome photonic lattice is experimentally realized under the EIT condition of a three-level Λ-type atomic system. In the experiment, the Kagome lattice with flatband and the one-dimensional periodic coupling field introduced to give rise to the type-III Dirac cones are all established by a SLM. The two coupling fields are injected into the ^85^Rb vapor cell with different spatial arrangements to modulate the refractive index at A, B, or C sublattices, and three different diffraction patterns can be observed at the output plane of the atomic vapor cell around two partially overlapped EIT windows. The generated type-III Dirac cones can be modulated easily via the laser parameters. The current work has provided an easy experimental way to prepare instantaneously reconfigurable photonic structures with type-III Dirac cones due to the inherited tunability from coherent multi-level atomic configurations. By taking advantages of such reconfigurability, it’s promising to uncover the rich underlying beam dynamics in type-III Dirac photonic structures in the future. Additionally, our study also extends the study of Dirac physics in electromagnetically induced photonic lattices from traditional type-I to type-III Dirac cones.

## Data Availability

Data underlying the results presented in this paper are not publicly available at this time but may be obtained from the authors upon reasonable request.
